# Studying the consumption and health outcomes of fiscal interventions (taxes and subsidies) on food and beverages in countries of different income classifications; a systematic review

**DOI:** 10.1186/s12889-015-2201-8

**Published:** 2015-09-14

**Authors:** AMAAP Alagiyawanna, Nick Townsend, Oli Mytton, Pete Scarborough, Nia Roberts, Mike Rayner

**Affiliations:** Ministry of Health, Health Education Bureau, No 2, Kynsey Road, Colombo, Sri Lanka; British Heart Foundation Centre on Population Approaches for Non-Communicable Disease Prevention, Nuffield Department of Population Health, University of Oxford, Old Road, Headington, Oxford, OX3 7LF UK; Centre for Diet and Activity Research (CEDAR), MRC Epidemiology Unit, University of Cambridge School of Clinical Medicine, Cambridge, CB2 0QQ UK; Bodleian Health Care Libraries, University of Oxford, Old Road, Headington, Oxford, OX3 7LF UK

## Abstract

**Background:**

Governments use fiscal interventions (FIs) on food and beverages to encourage healthy food behaviour and positive health outcomes. The objective of this review was to study the behavioural and health outcomes of implemented food and beverage FIs in the form of taxes and subsidies in countries of different income classifications.

**Methods:**

The present systematic review was conducted in accordance with Cochrane protocols. The search was carried out on academic and grey literature in English, for studies conducted in different countries on implemented FIs on food and non-alcoholic beverages and health outcomes, with a special focus on the income of those countries.

**Results:**

Eighteen studies met the inclusion criteria and 14 were from peer- reviewed journals. Thirteen studies came from high-income (HI) countries, four from upper middle-income (UMI) countries and only one came from a lower middle-income (LMI) country. There were no studies from lower-income (LI) countries. Of these 18 studies; nine focused on taxes, all of which were from HI countries. Evidence suggests that FIs on foods can influence consumption of taxed and subsidized foods and consequently have the potential to improve health.

**Conclusion:**

Although this review supports previous findings that FIs can have an impact on healthy food consumption, it also highlights the lack of evidence available from UMI, LMI and LI countries on such interventions. Therefore, evidence from HI countries may not be directly applicable to middle-income and LI countries. Similar research conducted in middle and low income countries will be beneficial in advocating policy makers on the effectiveness of FIs in countering the growing issues of non-communicable diseases in these countries.

**Electronic supplementary material:**

The online version of this article (doi:10.1186/s12889-015-2201-8) contains supplementary material, which is available to authorized users.

## Background

Changes in diet and physical activity, towards less healthy behaviours, are fuelling the rising obesity levels in LI countries [1]. This burden along with growing evidence on the causal relationship between unhealthy diet and increased non-communicable disease risk, has led to renewed emphasis on public health strategies aimed at improving dietary behaviour. One such strategy which has gained considerable attention is the use of targeted taxes and/or subsidies to influence food consumption [2–9]. The World Health Organization (WHO) has considered economic tools, such as these, to discourage the consumption of less healthy options and to improve the consumption of healthier food products by increasing accessibility, availability and affordability [10, 11].

Governments commonly use taxation and subsidies as FIs to encourage healthy food behaviours apart from the direct provision of certain health services at free or at subsidized rates. Examples of such fiscal policies include taxes levied on tobacco and alcohol and taxes on unhealthy foods, such as sugar sweetened beverages, along with subsidies on healthy foods which are thought to encourage more healthy purchasing and promote dietary behaviour [12].

To date the evidence for the effectiveness of FIs targeting food and beverages comes from natural experiments, controlled trials and modelling studies, although it is far from complete. The majority of systematic reviews targeting FIs and health outcomes focus on these approaches, with no published systematic review focusing only on the effectiveness of implemented food and beverage FIs in improving health.

The volatility of food prices and consumer responses to food taxes may be quite different in low and middle income countries compared to HI countries. A number of systematic reviews have concluded that taxes and subsidies on food can have favorable effects on diet [13–20]. Although there are systematic reviews which take into account research from countries of different income classifications, these reviews have paid little attention to differentiate these countries when studying the effectiveness of such interventions [13].

Seven recent review articles are particularly relevant to the present review. Wall et al. [20], Thow et al. [13] and An [14] carried out global reviews on the effectiveness of FIs in 2006, 2010 and 2013 respectively. Wall et al. [20] included only randomized controlled trials (RCTs) in their review and concluded that dietary behaviour could be influenced by monetary incentives. Thow et al. [13] reviewed empirical and modelling studies of the effectiveness of FIs on specific food products, on consumption and health outcomes. They concluded that food taxes and subsidies can influence dietary behaviour in HI countries and health outcomes could be improved by substantial FIs [13]. An [14] reviewed field experiments performed on food subsidies and concluded that subsidies could be effective in changing dietary behaviour. Eyles et al. [15] included modelling studies from countries in the Organization for Economic Cooperation and Development. They concluded that beneficiary dietary change, with the potential for improving health, could be achieved by taxing carbonated drinks and saturated fats [15]. Powell et al. [17] and Powell and Chaloupka [18] included only USA studies. Powell et al. [17] focused on price elasticity of demand studies studying the effectiveness of FIs on demand and body weight (BW) outcomes, finding that reducing obesity among lower socioeconomic groups may be achieved by reducing the cost of fruits and vegetables (F&V) through subsidies. Powell and Chaloupka [18] found that nontrivial pricing interventions may have some measurable weight outcomes, especially among children and adolescents. Black et al. [19] reviewed the effectiveness of food subsidy programmes on disadvantaged families in HI countries. They found that when the dietary changes are sustained, the rate of non-communicable diseases in adults could be reduced by improving intake of targeted nutrients and foods.

The present systematic review has taken into account international evidence, from countries with different income classifications, on the effectiveness of implemented food and beverage taxes and subsidies on consumption and health outcomes.

## Methods

The present review was undertaken based on the methods outlined in the Cochrane Handbook [21] and the Cochrane Health Promotion and Public Health Guidelines [22], in order to answer the following research questions: (i) Is there evidence of an effect of implemented food and beverage taxes and/or subsidies on behavioural or health outcomes? (ii) Does the evidence of an effect of these differ between countries of different income groups, as determined by the World Bank?

### Criteria for inclusion

#### Study design

Controlled and non-controlled trials, interrupted time- series (ITS) analysis of routine data, cross sectional, cohort and case control studies were eligible for inclusion. ITS of routine data were defined as analyses where data had been collected at three or more points, with at least one time point before and at least one time point after an intervention was implemented.

Eligible participants were both adults and children.

#### Types of interventions

Empirical studies which examined the implemented FIs at the national or local level were included. Empirical studies were defined as those that examined the effect of actual FIs. Both academic and grey literature were included. Grey literature is defined as academic literature that is not formally published [23]. Taxes on specific food products, such as increases in the cost of soda drinks and vending machine products, were included. Subsidy types included price discounts and vouchers for healthy foods. Emergency food relief services and general agricultural subsidies were excluded as they provide intermittent or one-off assistance which are unlikely to produce sustained impacts on food intake and have different aims to traditional FIs.

#### Outcomes

For inclusion, a study must have reported validated measures of at least one of the following health or behavioural measures as a primary outcome: (i) anthropometric measurements, e.g., body mass index (BMI), waist circumference, height for age (HA) (ii) nutrient intakes (iii) any health outcomes related to diet e.g., mortality, morbidity, hospital attendance/admissions (iv) pregnancy-related outcomes, e.g., low birth weight.

In addition to the above outcomes, other impacts of the tax or subsidy mentioned by the paper were also recorded, such as revenue loss, dependency on subsidies, decreased total food expenditure and increased intake of high fat or high-sugar foods.

#### Search strategy

**Fig. 1:**
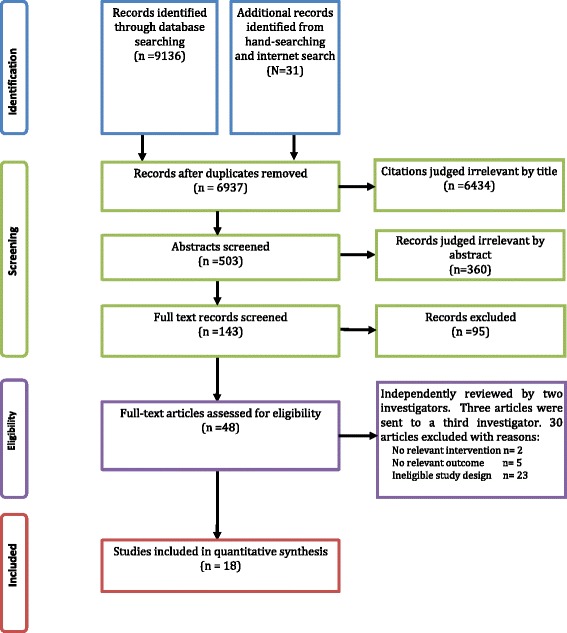
Flow chart of search results

The search strategy used a number of databases (Table 1), included English language literature from the earliest publication date to July 2013. We employed combinations of free-text and thesaurus search terms to describe the key concepts of food taxes/subsidy, food consumption and LMICs, full details are available in Additional file 1. We also conducted a reference list search of 14 reviews [13, 15–20, 24–30] yielding 1 further publication [31], which was not found using our search strategy. Finally, 18 studies were included in the current review. The detailed screening process is illustrated in Fig. 1.

**Table 1:** Databases used in search strategy

Medline (OvidSP) [1946-present], PubMed, EconLit and PAIS (Proquest), Global Health (OvidSP) [1973-present], Global Health Library:
http://www.globalhealthlibrary.net/php/index.php, Dissertations & Theses (Proquest), Science Citation Index, Social Science Citation Index, Conference Proceedings Citation Index – Science & Conference Proceedings Citation Index – Social Science & Humanities (Web of Science, Thomson Reuters)[1945-present], OECDiLibrary, ClinicalTrials https://clinicaltrials.gov/, OPENGrey http://www.opengrey.eu/, www.google.com, using Google to search specific web-sites: OECD, World Bank, WHO.org Sites & Org.uk. We used the Thomson Research Soft Endnote 5 present review [54].

#### Ethical statement

An ethics review was not required for this work.

#### Data synthesis and analysis

All manuscripts were downloaded into an Endnote library. A standardized data extraction form was used to collect the following variables from each included study: economic group listed by the World Bank in 2012 [32], type of FI (tax or subsidy), author, year of the publication and location of study, study type, period of study, duration of intervention, nature of tax or subsidy, outcome measure, study population, sample size, outcome data source, major findings, other effects, academic or grey literature and study quality. All the data extracted were checked by two researchers, a third was consulted for disputed inclusion.

### Assessment of the risk of bias in included studies

The quality assessment was conducted using the Evaluation of Public Health Practice Projects (EPHPP) [33] which is a standardized evaluation framework. This tool assesses six methodological dimensions: selection bias, study design, confounders, blinding, data collection methods, and withdrawals and dropouts, all of which feed into the calculation of a global rating. Each dimension is rated on a three-point scale: strong, moderate, or weak. The EPHPP tool was created primarily for individual level observational and clinical studies based on populations. Rating criteria for some items were modified by authors to improve the suitability of the tool for the population level interventions included in this review. These criteria are described in Additional file 2.

## Results

The systematic review process was divided into four major steps (identification, screening, eligibility and inclusion) according to the PRISMA statement [34]. The structured literature search identified 6937 potentially relevant citations after removing duplicates. Of these, 503 potentially relevant abstracts were screened: 360 records were excluded yielding 143 eligible records. Forty eight full text manuscripts for potentially eligible studies were assessed by two authors (AA and NT) for eligibility criteria. Since there were discrepancies between the two authors for three articles, those manuscripts were sent to a third reviewer (OM) and consensus achieved. Finally, 18 articles (Fig. 1) (17 separate studies including 14 peer-reviewed studies) met the inclusion criteria and were included in the review [31, 35–51]. The majority (13/18) of studies came from HI countries, 4 from UMI and one from a LMI country. No studies were found from LI countries. Of these 18 studies; 9 (50 %) focused on taxes, all of which were from HI countries. No studies included both taxes and subsidies together, although the LMI study did consider price elasticity alongside a food subsidy programme. Tables 2, 3 and 4 show the characteristics and distribution of studies based on behavioural/health outcomes of FIs according to the World Bank list of economies 2012 [32]. The date of publication ranged from 1990 to 2013, with 13 out of 18 published in 2008 or later.

**Table 2:** Characteristics of studies on food and beverage taxation in high-income countries

Study, year & location	Study type, study period & intervention period	Nature of tax or subsidy	Outcome measure	Study population	Sample size	Outcome data source	Major findings	Other impacts	Peer reviewed	Study quality
Bahl [48] 2003 Ireland	Natural experiment	Excise tax on soft drinks decreased from IR£ 0.37/gal in 1980–1990 to IR£ 0.29/gal in 1990–1992	Soft drink consumption data	Total population	Not specified	Soft Drink Association of Ireland	Soft drink consumption increase was 6.8 % between 1990 and 1992	Revenue loss approximately IR£ 2 million/year	Yes	Moderate
	1975–1996									
	21 years									
Oaks [49] 2005 State of Marine, USA	Interrupted time series with a control group	State tax of 5.5 % on soft drinks and selected snacks	BMI	Adults in Maine	Not specified	Behavioural Risk Factor Surveillance system (BRFSS)	No association between obesity and state tax	None recorded	No	Strong
	1991–2001									
	8 years									
Kim [43] 2006 USA	Cross sectional study	State level taxes on soft drinks or snacks	State level obesity prevalence	Total population	Not specified	BRFSS	No association between soft drink taxes and the obesity.	None recorded	Yes	Moderate
	1991–1998						States that repealed soft drink tax were 13 times more likely to have a high relative increase in obesity prevalence (defined as 75th percentile in the relative increase OR = 13.3; 95 % CI =0.7 – 272.0, *p* = 0.09) compared to states with a tax.			
	8 years									
Fletcher [42] 2010 USA	Cross sectional study	Mean soft drink tax rate among states with a tax between 4.1–5.1 %.	Soft drink and other beverage consumption, BMI, obesity, overweight	Children and adolescents in the USA	*n* = 22,132	National Health Examination and Nutrition Survey (NHANES)	1 % point increase in the soft drink tax rate resulted in a reduction in the daily consumption of soft drinks by 18 g (*p* < 0.05).	Whole milk as a substitute for soft drinks; a 1 % point increase in the soft drink tax rate increased whole milk consumption by 11.1 g per day (*p* < 0.001)	Yes	Weak
	1989–1994 1999–2006						Reduction in consumption of soda is completely offset by increase in consumption of other high-calorie drinks.			
	15 years						No association between soft drink taxes and BMI, obesity, overweight (*p* > 0.05).			
Powel [35] USA 2009	Longitudinal study	State-level carbonated soda sales tax range 0–8 %	BMI	8th, 10th and 12th grade students (13 – 19 years of age).	*n* = 153,673	Monitoring the Future Survey	No association between taxes and obesity among adolescents at state level.	None recorded	Yes	Moderate
	1997–2006						Small weakly statistically significant (*p* < 0.1) negative association was found between vending machine soda tax rates and BMI (−0.006) among teens at risk for overweight (*p* = 0.09).			
	10 years									
Fletcher [36] 2010 USA	Cross sectional study	State - level soft drink taxes. Range of mean total tax 3.3 – 5.0 %	BMI	Age ≥18 years in the USA	*n* = 2,709,422	BRFSS	1 % point increase in state soft drink tax rate leads to a decrease in BMI of 0.003 points (*p* < 0.01) and a decrease in obesity and overweight of 0.01 % (*p* < 0.1) and 0.02 % (*p* < 0.01) percentage points respectively.	None recorded	Yes	Moderate
	1990 – 2006						The impact of state soft drink taxes is larger for females, middle-aged and older individuals, individuals with greater education, and varies according to race and ethnic categories.			
	16 years									
Fletcher [47] 2010 USA	Cross sectional comparison study	Mean soft drink tax rate among states with a tax 4.7 %	Soft drink consumption, BMI	Children and adolescents in the USA	*n* = 20,968	NHANES	Soft drink tax was not effective at reducing soft drink consumption or BMI.	None recorded	Yes	Moderate
	1988–1994 1999–2006									
	15 years									
Nicholson [50] 2010 USA	Cross sectional comparison study	State level fast food restaurant and soda taxes	BMI	Adults 20–64 years of age	*n* = 1,948,833	BRFSS	High tax rate (≥8 %) in fast food restaurants significantly reduce mean BMI (−0.55) among females (*p* < 0.05).	None recorded	No	Moderate
	1997–2008						Soda tax did not significantly change BMI for all individuals.			
	12 years									
Sturm [46] 2010 USA	Cross sectional study 2004 1 year	State level carbonated soda sales tax is 4.2 %	Soda consumption, BMI	Children in 5^th^ grade students	*n* = 7300	Early Childhood Longitudinal study - Kindergarten cohort 2004	Soft drink taxes did not significantly affect overall levels of soda consumption or obesity rates.	None recorded	Yes	Moderate
							Higher soda taxes were associated with significantly lower (*p* < 0.05) BMI gain (−0.033) for the heavier children. Higher soda taxes were associated with significantly lower (*p* < 0.05) consumption (− 0.165 soda drinks per week) at school for the heavier children.

**Table 3:** Characteristics of studies on food and beverage subsidies in high-income countries

Study, year & location	Study type, study period & intervention period	Nature of tax or subsidy	Outcome measure	Study population	Sample size	Outcome data source	Major findings	Other impacts	Peer reviewed	Study quality
Currie [31] 2008 California, USA	Interrupted time series	Standard FSP –monthly food vouchers for any foods up to $142 per households per month dependent on income	Median birth weight, % of low birth weight, fetal survival	Pregnant women	*n* = 4,864,673	Data on FSP participation from annual state,	FSP had a statistically significant (*p* < 0.05) but very small effect on the probability of fetal survival in Los Angeles amongst whites only (0.01 % greater for infants between 1500 and 2000 grams), though no effect was seen in the state as a whole.	None recorded	No	Strong
	1961–1974 Duration of prenatal Food Stamp Programme (FSP) participation.					Forecasts of participation by county.	Introduction of FSP did not have any effect on low birth weight.			
						Individual birth records				
Herman [40] 2008 Los Angeles, California USA	Controlled before and after study	Standard Special Supplemental Nutrition Programme Women, Infants, and Children (WIC) programme plus $10 voucher weekly for Fruit and Vegetables (F&V) at two sites: 1) local supermarket, 2) farmer’s market.	F&V intake	Low-income postpartum women	intervention 1, *n* = 168, intervention 2, *n* = 140; control *n* = 143	WIC	Participants in the intervention sites increased consumption of F&V. The increase was sustained 6 months after the intervention was terminated (*p* < 0.001).	None recorded	Yes	Weak
	2001						Farmers market participants increased consumption of F&V by 1.4 servings per 100 kcal of consumed food (*p* < 0.001) from baseline to the end of intervention compared to the control group, and supermarket participants increased by 0.8 servings per 100 kcal (*p* = 0.02)			
	6 months									
Baum [41] 2012 USA	Longitudinal study	FSP on expectant mothers	Weight gained by expectant mothers during pregnancy	Low income expectant mothers	*n* = 709	National Longitudinal Survey of Youth 1979	Food Stamp Receipt (FSR) decreases the probability of gaining insufficient weight during pregnancy with FSR increasing pregnancy weight gain by 1.78 lb (*p* < 0.05). However, it does not result in pregnant mothers gaining too much weight	None recorded	Yes	Weak
	1979–2002									
	23 years									
Black [45] 2013 New South Wales, Australia	Before and after uncontrolled study	Weekly box of subsidized fruit and vegetables up to $60 linked to preventive health services (annual health assessment including dental and hearing check-ups, blood testing) and nutritional promotion	Change in the episode of illness, health service and emergency department attendances, antibiotic prescription, BMI	Low-income Aboriginal families with one or more childre*n* <17 years of age who were regular patients at the respective health services	*n* = 167	Retrospective health records audit and health assessment from Aboriginal health services, local hospitals and any other nominated general practices	A significant decrease (*p* < 0.05) in oral antibiotics prescribed (−0.5 prescriptions/year; 95%CI, −0.8 to −0.2) during 12 months of participation in the programme compared with the 12 months before the programme.	None recorded	Yes	Weak
	2008–2010						No significant reduction of BMI.			
	2 years						Significant increase (*p* < 0.05) in mean Haemoglobin level (3.1 g/L; 95 % CI, 1.4–4.8 g/L).

**Table 4:** Characteristics of studies on food and beverages in middle income countries

Upper middle-income countries										
Study, year & location	Study type, study period & intervention period	Nature of tax or subsidy	Outcome measure	Study population	Sample size	Outcome data source	Major findings	Other impacts	Peer reviewed	Study quality
Musgrove [38] 1990 Brazil	Cross sectional comparison study	Two programmes distributed free foods while another two programmes subsidized four or more basic food stuffs	Infant and child weight for age, weight for height, birth weight	Infant and children, pregnant women and nursing mothers	*n* = 10,071 families	Pan American Health Organization and Brazilian public agencies	Programmes were observed to be more effective at curing than at preventing malnutrition, and more effective at increasing weight than height.	Up to the end of 1986 the government cost was $767 million	Yes	Weak
	1974–1986						Many beneficiaries even when initially underweight, showed no change, and some deteriorated despite the food transfer.			
	12 years									
Sampaio [51] 1991 Recife, Brazil	Controlled before and after study	20 % food -price subsidies for 11 commodities	Consumption of 11 subsidized commodities, percentage of children with low birth weight, children’s nutritional status	Children under 5 years with low birth weight	intervention *n* = 100, control *n* = 100	PROAB data	PROAB programme may have small effect on calorie consumption but little or no effect on nutrition status and weight at birth	None recorded	No	Weak
	1987									
	9 years									
Osberg [44] 2009 Nine provinces in China	Cross sectional comparison	Food coupons for the purchase of rice, flour, and cooking oil at below market prices. The subsidy rate was 16.5 % of the income of a three person family living at US$ 2 per day.	Height for age	Chinese children aged 2–13 years	1991–1993 *n* = 1230, 1993 – 1997 *n* = 638, 1997 – 2000 *n* = 583	China Health and Nutritional Survey data	Food coupon use in earlier period correlates positively (*p* < 0.1) with growth in height-for-age.	Poverty was correlated with slower growth in height for age between 1997 and 2000 but not earlier. Poverty was negatively correlated with strong growth in height-for-age in 2000	Yes	Weak
	1991–2000	1991–1993 food subsidies were initially in place, 1993–2000 food subsidies had largely been abolished.								
	10 years									
An [39] 2013 South Africa	Cross sectional comparison	Up to 25 % discount on selected food items in about 800 supermarkets	Consumption of healthy foods, BMI	Health insurance plan members	*n* = 351,319	Health Risk Assessment Survey	A 10 % and 25 % discount on healthy food is associated with: an increase in daily fruits and vegetable consumption by 0.38 (*p* < 0.001) and 0.64 (*p* < 0.001) servings respectively; having ≥ 3 servings of wholegrain food daily by 2.05 (*p* < 0.001) and 2.96 (*p* < 0.001) respectively; but less likely to regularly have foods high in sugar with an OR of 0.59 (*p* < 0.001) and 0.26 (*p* < 0.001), fried foods with an OR of 0.53 (*p* < 0.001) and 0.26 (*p* < 0.001), processed meats with an OR of 0.71 (*p* < 0.001) and 0.33 (*p* < 001), and fast food with an OR of 0.54 (*p* < 0.001) and 0.28 (*p* < 0.001) respectively.	None recorded	Yes	Weak
	2009–2011						There was no strong evidence that participation in the Healthy Food Programme reduced BMI but there is a statistically significant (*p* < 0.001) relationship between 25 % discount on healthy food purchases and obesity with an OR of 0.86 (95 % CI 0.81–0.91).			
	3 years									
Lower middle-income countries										
Asfar [37] 2007 Egypt	Ecological study 1997 1 year	Food subsidy programme: 57 % for bread; 42–62 % for sugar	Mother’s BMI	Pregnant mothers in Egypt	Individual *n* = 902, household *n* = 2000 from 20 governorates.	Egyptian Integrated Household Survey	The subsidy programme pushed people towards obesity.	Cost US$ 1.1 billion in 1997	Yes	Weak
							There was an inverse and statistically significant (*p* < 0.05 %) relationship between mother’s BMI and the price of baladi bread and fully and partially subsidized sugar. There is a direct and statistically significant (*p* < 0.05 %) relationship between high quality but expensive foods, like fruits, milk and egg and BMI of mothers.			

In HI countries statistically significant findings were reported between subsidies and “F&V intake” [40], maternal weight gain [41], reduction in antibiotic prescriptions [45] and increase in mean haemoglobin levels [45]. Subsidies were not associated with “BMI” [45], low birth weight or fetal survival [31].

Subsidies were also found to be associated with consumption of healthy foods [39], increase in HA [44] in UMI countries, with two further studies [38, 51] reporting effects on calorie intake and malnutrition but not presenting any significance testing. One study found no association between subsidies and BMI [39]. The LMI country study reported that a subsidy programme pushed people towards obesity.

All studies on the impact of taxes came from HI countries. A link was found between soft drink tax and consumption by children and adolescents [42] and subgroups of at-risk children, including those who are already overweight, come from low-income families, or are African American [46]. Another study reported increases in consumption of soft drinks due to tax reduction but presented no significance testing [48]. Two studies found no association between soft drink tax and consumption [46, 47]. One study reported that soft drink tax could influence BMI [36] whilst another study found a weakly negative association between soda tax and BMI among teens at risk of overweight [35]. Six studies found no impact on obesity/BMI [35, 42, 43, 46, 47, 49], a further two reported an impact on the BMI of heavier children [46] and females [50] due to higher tax rate.

Findings from all these studies are described in more detail according to the income classification of their country of study below.

### High income countries

#### Effect on consumption

Two peer-reviewed studies from HI countries found that change to a tax/subsidy altered consumption in the expected direction. In an empirical study in Ireland, Bahl et al. [48] found a 20 % reduction in soft drink tax resulted in a 6.8 % increase in average soft drink consumption.

Herman et al. [40] assessed the impact of an additional US$10/week on F&V subsidy for standard Special Supplementation Nutrition Programme for Women, Infants, and Children, in the US in a controlled before and after (CBA) study. Participants in the intervention sites increased consumption of F&V. The increase was sustained 6 months after intervention was terminated (*p* < 0.001).

#### Effect on anthropometry

Three studies from the USA [35, 36, 43] studied the impact of a tax or subsidy on BW/BMI. Two of these studies [35, 43] examined the association between soda taxes and BW. One study found no cross-sectional association between state-level taxes (in the range 0–8 %) and BW, although a non-significant trend towards a small increase in obesity prevalence among states with a tax was observed [43]. A second study found no association between state-level taxes (range 0–8 %) and adolescent weight overall, although a weak effect was observed between taxes and overweight in adolescents [35]. Fletcher et al.[36] studied the relationship between state-level soft drink taxes (3 %) in the USA and population BMI among adults aged 18 years and over between 1990 and 2006. They found that even relatively large tax increases had little effect.

Two studies investigating the effect of taxes on BMI were identified from the grey literature. Oaks [49] found no association between obesity prevalence and a snack and soft drink tax of 5.5 % in Maine, USA, on comparing obesity rate over 15 years with that in New Hampshire, a state with no tax. Nicholson [50] analysed state level soda taxes in USA among a sample of adults but did not find a relationship with BMI, however, higher taxes (>8 %) in fast-food restaurants was significantly associated with reduction in BMI (−0.55) among females (*p* < 0.05).

#### (PRO)

Currie and Moretti 2008 [31] examined the Food Stamp Programme (FSP) in the USA, which subsidized up to $142 per household, on PRO. They carried out an ITS analysis of births in California and found no significant changes overall in low birth weight after the introduction of the FSP in the 1960s. This study was published in grey literature

In a longitudinal study in the USA, Baum 2012 [41] found that food stamp receipt (FSR) decreased the probability of gaining insufficient weight during pregnancy, with FSR increasing pregnancy weight gain by 1.78 lb (*p* < 0.05). However, it didn’t increase the probability of expectant mothers gaining too much weight during pregnancy.

#### Effect on consumption and anthropometry

Three peer-reviewed studies from the USA investigated the effect of tax/subsidies on consumption and BW. Fletcher et al. [42, 47] reported the effect of soft drink taxes on consumption and BMI of children aged 3–18 years in two articles. Both articles reported that a mean soft drink tax of around 4.5 % was not associated with a change in BMI. The earlier paper [42] reported a moderate reduction of soft drink consumption, but that this was offset by the increase in consumption of other high-calorie drinks. The latter paper [47] reported no evidence of an effect. Sturm et al. [46] examined the effect of state level carbonated soda tax (4.2 %) on kindergarten students. They found no evidence of an associated change in consumption or BMI.

#### Effect on health outcomes related to diet and anthropometry

A study from Australia [45], examining the health effect of a F&V programme subsidizing up to $60/week, looked at six main health outcomes: change in illness episode, health service and emergency department attendances, antibiotic prescription, anthropometry, haemoglobin and iron status. They found no association with BMI but a significant decrease (*p* < 0.05) in oral antibiotics prescribed and a significant increase (*p* < 0.05) in mean haemoglobin level (3.1 g/L; 95 % CI, 1.4–4.8 g/L).

### Upper middle income countries

#### Effect on anthropometry

Osberg et al. 2009 [44] examined the effect of a food coupon programme in China, providing subsidies of up to 16.5 % on rice, flour, and cooking oil for families of children below the expected HA. They found a positive (*p* < 0.1) correlation between the subsidy and growth in HA between 1997 and 2000. In 2000, poverty was negatively correlated with strong growth in HA.

#### Effect on consumption and anthropometry

An et al. [39] examined the effect of subsidised (up to 25 % discount) healthy food on consumption and BMI in South Africa (a UMI country), they found a significant increase in consumption of subsidized food but no association with BMI. They reported that programme participation was associated with higher consumption of F&V (*p* < 0.001) and wholegrain food (*p* < 0.001); and lower consumption of high sugar food (*p* < 0.001), fried food (*p* < 0.001), processed meat (*p* < 0.001) and fast-food (*p* < 0.001). There was no strong evidence that participation reduces obesity but there was a statistically significant relationship between a 25 % discount on healthy food purchases and lower BMI (*p* < 0.001)

#### Effect on anthropometry and PROs

Musgrove [38] examined four nutrition programmes in Brazil. Two programmes distributed free foods while another two programmes subsidized four or more basic foodstuffs. One of the subsidized programmes was a quantitatively restricted subsidy provided to unidentified families; the other was unrestricted and open to all families using certain shops. The study was published in a peer-reviewed journal and reported no effect of food subsidies on anthropometry or PRO.

#### Effect on consumption, PROs and nutritional status

One study [51] from a UMI country (Brazil) examined the effect of 20 % food price subsidies for 11 commodities, finding a small increase in calorie consumption but little or no effect on nutrition status and birth weight. This was published in grey literature.

### Lower middle income countries

#### Effect on anthropometry

Asfaw 2007 [37] assessed the direct effect of subsidies on four food items ranging from 42 to 62 % in Egypt (LMI country) on BW using historical data on price and consumption. The findings from this study suggest that reducing subsidies to create a 1 % increase in bread and sugar prices would reduce the average BMI of mothers in the country by 0.12 and 0.11 % respectively.

### Quality assessment

The quality assessment of studies was conducted using the EPHPP tool, adapted to be suitable for use on studies concerning population FIs. A lower rating may not be a reflection of a weak study in itself, but indicates a lower rating in suitability for our purposes; evaluating the impact of FIs on a population level. An overall rating is added to Tables 2, 3 and 4, a full breakdown of ratings by category can be found in Additional file 3. Nine studies were evaluated as weak [37–42, 44, 45, 51] in the global rating, seven studies were evaluated as moderate [35, 36, 43, 46–48, 50] while two studies were evaluated as strong [31, 49]. All studies from UMIs and LMIs were graded as weak.

## Discussion

The present review found that taxes and subsidies are associated with changes in dietary behaviour [39, 40, 42]. However, there is only limited evidence on how these changes in dietary behaviour translate into detectable health outcomes. Some studies [35, 36, 42, 46, 47, 49, 50] have indicated that the taxes implemented so far have been too low to have a detectable effect on health outcomes. There is also some empirical evidence [42] to suggest that taxation has led to substitution of unhealthy products.

This review has a number of limitations. As we only considered studies on implemented FIs, taxes were often of a low level and much evidence came from cross-sectional studies, which do not allow an inference of causality. As the included studies differed substantially by study population, intervention setting, study design and outcome measures, data sources and analytical methods, it is difficult to compare the effectiveness of FIs on diet and health outcomes. No RCTs were found, which probably reflects the difficulty of implementing such interventions on this basis, including the ethical issues arising when studying at a population level. Data on dietary behaviour was based on self-report, which has well recognized limitations due to imprecision and potential recall biases [52]. Additionally we were unable to identify a tool designed to assess the quality of studies on implemented population level interventions, we adapted the EPHPP, a tool primarily designed for individually-focused studies, to make it more applicable for this review. A further limitation of the present review is that only English language literature was included.

One of the key strengths of the current review is that it was carried out according to the protocols recommended by the Cochrane Collaboration [21]. The present review is also broader in scope than previous reviews [17, 18, 20] as it included both peer reviewed and grey literature from the earliest publication date to July 2013. Grey literature was important for identifying additional papers, in particular those from LI countries. The rigorous review process for selecting the manuscripts and extracting the data, including both the effectiveness and quality assessments, is also a strength. To our knowledge, this is the first systematic review that considers the global evidence on the effect of implemented food and beverage FIs, with a specific focus on comparing between countries of different income classification. The review was reported transparently using the PRISMA checklist (please see Additional file 4).

Previous reviews on the dietary and health outcomes due to FIs have focused on modelling studies [15], both modelling and empirical studies [13] and controlled trials [14, 19, 20]. Modelling studies and experiments in closed environments have limitations. However, empirical studies should provide more robust results than predictive studies. Empirical studies that used regression analysis to assess the link between taxes and obesity were also methodologically weak because consumption of the taxed foods was not measured, making it difficult to determine whether change in taxes caused the observed weight changes. Thus, the overall quality of the evidence is limited and the findings should be interpreted with caution.

The present review confirms the findings of previous reviews [13–15, 20] that taxes and subsidies on food could modify dietary behaviour and health outcomes. Similar to previous reviews [13, 14, 20], this review also focused on global evidence. Wall et al. [20] restricted their review to include RCTs only. Limiting the scope to include only those studies that are conducted in highly controlled environments, make it difficult to study the real life dynamics of introducing FIs. An [14] studied the effectiveness of field experiments and found that food subsidies are effective in changing dietary behaviours. In LMI and LI countries, subsidies are the most prominent type of FIs. However, the findings of An [14] only found one study that wasn’t from a HI country and none from LMIs or LIs, therefore findings cannot be directly attributed to these countries as the social and financial context of the settings are different. In addition the value of studying subsidies on their own is limited as most studies describe a synergistic effect in using taxation and subsidies in combination. Thow et al. [13] also found positive food consumption behaviour linked to FIs in empirical and modelling studies conducted in HI countries. Powell et al. [17], Powell and Chaloupka [18] and Black et al. [19] focused on studies from HI countries, finding positive behaviour changes in food practices were associated with FIs. The challenge from this evidence is in identifying how it can be translated into action in LMI and LI countries.

Knaul and Nugent 2006 [53] highlight the problems of implementing FIs in LMI and LI countries. The priorities of LI countries differ significantly from those of HI countries, in terms of malnutrition. LI and LMI countries are more affected by under-nutrition, rather than over-nutrition, as commonly observed in HI countries. Furthermore, some LMI countries are affected by a double burden of malnutrition with under nutrition and over nutrition co-existing in the same population, making it difficult to introduce blanket interventions for the entire country. Any fiscal measures, either subsidies or taxes, may therefore have beneficial effects on one group of people but may also aggravate the conditions of others. They further emphasize how food subsidies have led to food smuggling and various other unlawful activities that have jeopardized the effectiveness of these FIs [53].

In UMI and LMI countries the evidence exclusively concerns subsidies, which can have a role in increasing calorie intake and consumption of healthy food items, where malnutrition is a concern. However, there is also a risk that subsidizing some high-calorie food products (e.g., bread and rice) may contribute to obesity [37]. FIs in the form of tax reductions and subsidies have been in effect in developing countries for a long period of time to ensure that adequate nutrition can be afforded by all citizens of the country. Even though some efforts have been taken to target these interventions at those who need it and exclude the affluent communities, by introducing ration cards, food stamps etc., success or failure of these is not well documented.

## Conclusions

There is evidence of the effectiveness of FIs in promoting behaviour change on a population level, so policy makers should consider these interventions. Although a number of FIs are being implemented in UMI, LMI and LIs, studies on taxes have been conducted only in HI countries. Similar research in UMI, LMI and LI countries would be beneficial in advocating policy makers to utilize FIs in countering the growing issues of NCDs in these countries. Interventions conducted in UMI, LMI and LIs can be different to those of HI countries and therefore further research should identify the types of FIs currently being implemented in these countries. This will add new knowledge to the global evidence base. Policy makers and researchers in all countries should go one step further to evaluate the impact of these on health outcomes and publish their findings to improve the quality of evidence on this topic.

## Additional files

Additional file 1:
**Search strategy.** (PDF 229 kb) Additional file 2:
**Adjusted criteria for the quality assessment tool for quantitative studies.** (PDF 88 kb) Additional file 3:
**Description of the intervention and quality assessment of study.** (DOC 106 kb) Additional file 4:
**PRISMA 2009 checklist.** (PDF 301 kb)
