# Supplementary material for: Studying the consumption and health outcomes of fiscal interventions (taxes and subsidies) on food and beverages in countries of different income classifications; a systematic review
Source: BMC Public Health. 2015 Sep 14;15:887. doi: 10.1186/s12889-015-2201-8 (PMC4570679; doi:10.1186/s12889-015-2201-8)
Supplement: Additional file 1: — Search strategy. (PDF 229 kb) [file 12889_2015_2201_MOESM1_ESM.pdf]

Additional file 1. Search strategy

Database:	Interface:	Dates:	Hits:
EconLit	Proquest	29/07/13	LMICs = 810<br>Other = 905
Global Health	OvidSP (1973-present)	29/07/13	LMICs = 841<br>Other =1224
Global Health Library	http://www.globalhealthlibrary.net/php/index.php	29/07/13	LMICs = 91
Grey literature web-sites	Sites listed in appendix	29/07/13 & 30/07/13	All =40
Medline	OvidSP (1946 – present)	29/07/13	LMICs = 541<br>Other =1760
PAIS International	Proquest	29/07/13	LMICs = 370<br>Other = 574
Dissertations & Theses	Proquest	29/07/13	LMICs = 113<br>Other = 292
Science Citation Index, Social Science Citation Index, Conference Proceedings Citation Index – Science & Conference Proceedings Citation Index – Social Science & Humanities	Web of Science (1945 – present)	29/07/13	LMICs = 419<br>Other =1156
Total:			9136
Duplicates:			2230
Animal studies removed:			91
Final total:			LMIC =1858<br>Other =4948

Limits applied:

English language

Additional search methods:

33 Systematic reviews were identified through a search of Medline (OvidSP), Cochrane Library and DARE.

Search strategy:

Global Health (OvidSP) [1973 – present]

1	(food* and (tax or taxes or taxing or taxation)).ti.
2	((beverage? or soda? or soft drink? or carbonated drink? or sugar sweetened drink?) and (tax or taxes or taxing or taxation)).ti.
3	((sugar* or fat or fats or sucrose or candy or sweet* or snack* or fastfood? or junkfood?) and (tax or taxes or taxing or taxation)).ti.
4	((diet* or nutrition* or obesity or obese or overweight) and (tax or taxes or taxing or taxation)).ti.
5	((vegetable? or fruit?) and (tax or taxes or taxing or taxation)).ti.
6	((vending machine? or takeaway?) and (tax or taxes or taxing or taxation)).ti.
7	(food* and (price? or pricing)).ti.
8	((beverage? or soda? or soft drink? or carbonated drink? or sugar sweetened drink?) and (price? or pricing)).ti.
9	((sugar* or fat or fats or sucrose or candy or sweet* or snack* or fastfood? or junkfood?) and (price? or pricing)).ti.
10	((vegetable? or fruit?) and (price? or pricing)).ti.
11	(food* and (subsidy or subsidies or incentiv* or voucher?)).ti.
12	((beverage? or soda? or soft drink? or carbonated drink? or sugar sweetened drink?) and (subsidy or subsidies or incentiv* or voucher?)).ti.
13	((sugar* or fat or fats or sucrose or candy or sweet* or snack* or fastfood? or junkfood?) and (subsidy or subsidies or incentiv* or voucher?)).ti.
14	((vegetable? or fruit?) and (subsidy or subsidies or incentiv* or voucher?)).ti.
15	(food* adj5 (tax or taxes or taxing or taxation)).ti,ab.
16	((beverage? or soda? or soft drink? or carbonated drink? or sugar sweetened drink?) adj5 (tax or taxes or taxing or taxation)).ti,ab.
17	((sugar* or fat or fats or sucrose or candy or sweet* or snack* or fastfood? or junkfood?) adj5 (tax or taxes or taxing or taxation)).ti,ab.
18	((vegetable? or fruit?) adj5 (tax or taxes or taxing or taxation)).ti,ab.
19	((diet* or nutrition* or obesity or obese or overweight) adj5 (tax or taxes or taxing or taxation)).ti,ab.
20	((vending machine? or takeaway?) adj5 (tax or taxes or taxing or taxation)).ti,ab.
21	(food* adj5 (price? or pricing)).ti,ab.
22	((beverage? or soda? or soft drink? or carbonated drink? or sugar sweetened drink?) adj5 (price? or pricing)).ti,ab.
23	((sugar* or fat or fats or sucrose or candy or sweet* or snack* or fastfood? or junkfood?) adj5 (price? or pricing)).ti,ab.
24	((vegetable? or fruit?) adj5 (price? or pricing)).ti,ab.
25	(food* adj5 (subsidy or subsidies or incentiv* or voucher?)).ti,ab.
26	((beverage? or soda? or soft drink? or carbonated drink? or sugar sweetened drink?) adj5 (subsidy or subsidies or incentiv* or voucher?)).ti,ab.

27 ((sugar* or fat or fats or sucrose or candy or sweet* or snack* or fastfood? or junkfood?) adj5 (subsidy or subsidies or incentiv* or voucher?)).ti,ab.

28 ((vegetable? or fruit?) adj5 (subsidy or subsidies or incentiv* or voucher?)).ti,ab.

29 or/1-28

30 foods/ or confectionery/ or convenience foods/ or desserts/ or fast foods/ or fried foods/ or health foods/ or food products/ or fruit products/ or sugar/ or vegetable products/ or exp vegetables/ or exp fruits/

31 feeding habits/ or feeding preferences/

32 beverages/ or fruit drinks/ or soft drinks/

33 food consumption/

34 food intake/

35 Food policy/ or Nutrition Policy/

36 (food* or beverage? or soda? or soft drink? or carbonated drink? or sugar sweetened drink? or sugar* or fat or fats or sucrose or candy or sweet* or snack* or fastfood* or vending machine*).ti.

37 (obese or obesity or overweight or diet* or nutrition*).ti.

38 or/30-37

39 taxes/ or direct taxation/ or indirect taxation/ or tax credits/ or tax incentives/

40 economic policy/ or fiscal policy/ or price policy/

41 food prices/ or prices/

42 subsidies/

43 (tax or taxes or taxing or taxation).ti.

44 ((price? or pricing or demand* or fiscal* or economic* or financ* or cost?) adj3 (elasticity or elasticities)).ti,ab.

45 ((price? or pricing or demand* or fiscal* or economic* or financ* or cost?) adj3 (policy or policies)).ti,ab.

46 ((price? or pricing or demand* or fiscal* or economic* or financ* or cost?) adj3 (priority or priorities)).ti,ab.

47 ((price? or pricing or demand* or fiscal* or economic* or financ* or cost?) adj3 (subsidy or subsidies or incentiv* or voucher? or reward?)).ti,ab.

48 or/39-47

49 38 and 48

50 ((food* or nutrition* or diet* or obes* or overweight) adj3 (policy or policies)).ti,ab.

51 (tax or taxes or taxing or taxation or price? or pricing or fiscal* or economic* or financ* or cost* or subsidy or subsidies or incentiv* or voucher?).ti,ab,hw.

52 50 and 51

53 29 or 49 or 52

54 (body mass or body fat* or bmi or obes* or overweight or body weight).af.

55 ((healthy or healthier or healthful or healthily) adj3 (food* or eating or choice* or lifestyle or lifestyle)).af.

56 (fruit* or vegetable*).af.

57 ((food* or energy or beverage* or drink*) adj2 (consumption or consume* or intake)).af.

58 (public health or healthy living or health promotion).af.

59 or/54-58

60 53 and 59

61 limit 60 to english language

62 exp developing countries/ or exp africa/

63 (Africa or Asia or Caribbean or West Indies or South America or Latin America or Central America).hw,ti,ab,cp.

64 ((developing or less* developed or under developed or underdeveloped or middle income or low* income or underserved or under served or deprived or poor*) adj (countr* or nation? or population? or world)).ti,ab.

65 (Afghanistan or Albania or Algeria or Angola or Antigua or Barbuda or Argentina or Armenia or Armenian or Aruba or Azerbaijan or Bahrain or Bangladesh or Barbados or Benin or Byelarus or Byelorussian or Belarus or Belorussian or Belorussia or Belize or Bhutan or Bolivia or Bosnia or Herzegovina or Hercegovina or Botswana or Brazil or Brasil or Bulgaria or Burkina Faso or Burkina Fasso or Upper Volta or Burundi or Urundi or Cambodia or Khmer Republic or Kampuchea or Cameroon or Cameroons or Cameron or Camerons or Cape Verde or Central African Republic or Chad or Chile or China or Colombia or Comoros or Comoro Islands or Comores or Mayotte or Congo or Zaire or Costa Rica or Cote d'Ivoire or Ivory Coast or Croatia or Cuba or Cyprus or Czechoslovakia or Czech Republic or Slovakia or Slovak Republic or Djibouti or French Somaliland or Dominica or Dominican Republic or East Timor or East Timur or Timor Leste or Ecuador or Egypt or United Arab Republic or El Salvador or Eritrea or Estonia or Ethiopia or Fiji or Gabon or Gabonese Republic or Gambia or Gaza or Georgia Republic or Georgian Republic or Ghana or Gold Coast or Greece or Grenada or Guatemala or Guinea or Guam or Guiana or Guyana or Haiti or Honduras or Hungary or India or Maldives or Indonesia or Iran or Iraq or Isle of Man or Jamaica or Jordan or Kazakhstan or Kazakh or Kenya or Kiribati or Korea or Kosovo or Kyrgyzstan or Kirghizia or Kyrgyz Republic or Kirghiz or Kirgizstan or Lao PDR or Laos or Latvia or Lebanon or Lesotho or Basutoland or Liberia or Libya or Lithuania or Macedonia or Madagascar or Malagasy Republic or Malaysia or Malaya or Malay or Sabah or Sarawak or Malawi or Nyasaland or Mali or Malta or Marshall Islands or Mauritania or Mauritius or Agalega Islands or Mexico or Micronesia or Middle East or Moldova or Moldovia or Moldovian or Mongolia or Montenegro or Morocco or Ifni or Mozambique or Myanmar or Myanma or Burma or Namibia or Nepal or Netherlands Antilles or New Caledonia or Nicaragua or Niger or Nigeria or Northern Mariana Islands or Oman or Muscat or Pakistan or Palau or Palestine or Panama or Paraguay or Peru or Philippines or Philipines or Phillipines or Phillippines or Poland or Portugal or Puerto Rico or Romania or Rumania or Roumania or Russia or Russian or Rwanda or Ruanda or Saint Kitts or St Kitts or Nevis or Saint Lucia or St Lucia or Saint Vincent or St Vincent or Grenadines or Samoa or Samoan Islands or Navigator Island or Navigator Islands or Sao Tome or Saudi Arabia or Senegal or Serbia or Montenegro or Seychelles or Sierra Leone or Slovenia or Sri Lanka or Ceylon or Solomon Islands or Somalia or Sudan or Suriname or Surinam or Swaziland or Syria or Tajikistan or Tadzhikistan or Tadjikistan or Tadzhik or Tanzania or Thailand or Togo or Togolese Republic or Tonga or Trinidad or Tobago or Tunisia or Turkey or Turkmenistan or Turkmen or Uganda or Ukraine or Uruguay or USSR or Soviet Union or Union of Soviet Socialist Republics or Uzbekistan or Uzbek or Vanuatu or New Hebrides or Venezuela or Vietnam or Viet Nam or West Bank or Yemen or Yugoslavia or Zambia or Zimbabwe or Rhodesia).hw,ti,ab,cp.

66 ((developing or less* developed or under developed or underdeveloped or middle income or low* income) adj (economy or economies)).ti,ab.

67 (low* adj (gdp or gnp or gross domestic or gross national)).ti,ab.

68 (low adj3 middle adj3 countr*).ti,ab.

69 (lmic or lmics or third world or lami countr*).ti,ab.

70 transitional countr*.ti,ab.

71 62 or 63 or 64 or 65 or 66 or 67 or 68 or 69 or 70

72 61 and 71
73 61 not 72
74 60 not 61

Global Health Library: http://pesquisa.bvsalud.org/ghl/index.php

(food* OR beverage* OR soda OR sodas OR soft drink* OR carbonated drink* OR sugar* OR fat OR fats OR sucrose OR candy OR sweet* OR snack* OR fastfood* OR junkfood* OR vending machine*) AND (tax OR taxes OR taxation OR taxing OR subsidy OR subsidies OR incentiv* OR voucher*) – in Regional Indexes

(obese OR obesity OR overweight OR fat OR "healthy eating" OR "healthy lifestyle") AND (tax OR taxes OR taxation OR taxing OR subsidy OR subsidies OR incentiv* OR voucher*) – in Regional Indexes

(food* OR beverage* OR soda OR sodas OR soft drink* OR carbonated drink* OR sugar* OR fat OR fats OR sucrose OR candy OR sweet* OR snack* OR fastfood* OR junkfood* OR vending machine*) AND ("price elasticity" OR "price elasticities" OR "deman elasticity" OR "demand elasticities" OR "financial elasticity" OR "financial elasticities" OR "pricing elasticity" OR "pricing elasticities" OR "economic elasticity" OR "economic elasticities" OR "cost elasticity" OR "cost elasticities" OR "fiscal elasticity" OR "fiscal elasticities") – in Regional Indexes

Medline (OvidSP) [1946-present]

1	(food* and (tax or taxes or taxing or taxation)).ti.
2	((beverage? or soda? or soft drink? or carbonated drink? or sugar sweetened drink?) and (tax or taxes or taxing or taxation)).ti.
3	((sugar* or fat or fats or sucrose or candy or sweet* or snack* or fastfood? or junkfood?) and (tax or taxes or taxing or taxation)).ti.
4	((diet* or nutrition* or obesity or obese or overweight) and (tax or taxes or taxing or taxation)).ti.
5	((vegetable? or fruit?) and (tax or taxes or taxing or taxation)).ti.
6	((vending machine? or takeaway?) and (tax or taxes or taxing or taxation)).ti.
7	(food* and (price? or pricing)).ti.
8	((beverage? or soda? or soft drink? or carbonated drink? or sugar sweetened drink?) and (price? or pricing)).ti.
9	((sugar* or fat or fats or sucrose or candy or sweet* or snack* or fastfood? or junkfood?) and (price? or pricing)).ti.
10	((vegetable? or fruit?) and (price? or pricing)).ti.
11	(food* and (subsidy or subsidies or incentiv* or voucher?)).ti.
12	((beverage? or soda? or soft drink? or carbonated drink? or sugar sweetened drink?) and (subsidy or subsidies or incentiv* or voucher?)).ti.
13	((sugar* or fat or fats or sucrose or candy or sweet* or snack* or fastfood? or junkfood?) and (subsidy or subsidies or incentiv* or voucher?)).ti.
14	((vegetable? or fruit?) and (subsidy or subsidies or incentiv* or voucher?)).ti.
15	(food* adj5 (tax or taxes or taxing or taxation)).ti,ab.
16	((beverage? or soda? or soft drink? or carbonated drink? or sugar sweetened drink?) adj5 (tax or taxes or taxing or taxation)).ti,ab.
17	((sugar* or fat or fats or sucrose or candy or sweet* or snack* or fastfood? or junkfood?) adj5 (tax or taxes or taxing or taxation)).ti,ab.
18	((vegetable? or fruit?) adj5 (tax or taxes or taxing or taxation)).ti,ab.
19	((diet* or nutrition* or obesity or obese or overweight) adj5 (tax or taxes or taxing or taxation)).ti,ab.
20	((vending machine? or takeaway?) adj5 (tax or taxes or taxing or taxation)).ti,ab.
21	(food* adj5 (price? or pricing)).ti,ab.
22	((beverage? or soda? or soft drink? or carbonated drink? or sugar sweetened drink?) adj5 (price? or pricing)).ti,ab.
23	((sugar* or fat or fats or sucrose or candy or sweet* or snack* or fastfood? or junkfood?) adj5 (price? or pricing)).ti,ab.
24	((vegetable? or fruit?) adj5 (price? or pricing)).ti,ab.
25	(food* adj5 (subsidy or subsidies or incentiv* or voucher?)).ti,ab.
26	((beverage? or soda? or soft drink? or carbonated drink? or sugar sweetened drink?) adj5 (subsidy or subsidies or incentiv* or voucher?)).ti,ab.
27	((sugar* or fat or fats or sucrose or candy or sweet* or snack* or fastfood? or junkfood?) adj5 (subsidy or subsidies or incentiv* or voucher?)).ti,ab.

28 ((vegetable? or fruit?) adj5 (subsidy or subsidies or incentiv* or voucher?)).ti,ab.

29 or/1-28

30 exp Food/

31 food habits/ or food preferences/

32 beverages/ or carbonated beverages/

33 Energy Intake/

34 Food Dispensers, Automatic/

35 Obesity/pc [Prevention & Control]

36 (food* or beverage? or soda? or soft drink? or carbonated drink? or sugar sweetened drink? or sugar* or fat or fats or sucrose or candy or sweet* or snack* or fastfood* or vending machine*).ti.

37 (obese or obesity or overweight or diet* or nutrition*).ti.

38 or/30-37

39 exp Taxes/

40 Health Policy/ec [Economics]

41 Nutrition Policy/ec [Economics]

42 (tax or taxes or taxing or taxation).ti.

43 ((price? or pricing or demand* or fiscal* or economic* or financ* or cost?) adj3 (elasticity or elasticities)).ti,ab.

44 ((price? or pricing or demand* or fiscal* or economic* or financ* or cost?) adj3 (policy or policies)).ti,ab.

45 ((price? or pricing or demand* or fiscal* or economic* or financ* or cost?) adj3 (priority or priorities)).ti,ab.

46 ((price? or pricing or demand* or fiscal* or economic* or financ* or cost?) adj3 (subsidy or subsidies or incentiv* or voucher? or reward?)).ti,ab.

47 or/39-46

48 38 and 47

49 ((food* or nutrition* or diet* or obes* or overweight) adj3 (policy or policies)).ti,ab.

50 (tax or taxes or taxing or taxation or price? or pricing or fiscal* or economic* or financ* or cost* or subsidy or subsidies or incentiv* or voucher?).ti,ab,hw.

51 49 and 50

52 29 or 48 or 51

53 limit 52 to english language

54 limit 53 to "reviews (maximizes specificity)"

55 Developing Countries/

56 (Africa or Caribbean or West Indies or South America or Latin America or Central America).hw,kf,ti,ab,cp.

57 (Afghanistan or Albania or Algeria or Angola or Antigua or Barbuda or Argentina or Armenia or Armenian or Aruba or Azerbaijan or Bahrain or Bangladesh or Barbados or Benin or Byelarus or Byelorussian or Belarus or Belorussian or Belorussia or Belize or Bhutan or Bolivia or Bosnia or Herzegovina or Hercegovina or Botswana or Brazil or Brasil or Bulgaria or Burkina Faso or Burkina Fasso or Upper Volta or Burundi or Urundi or Cambodia or Khmer Republic or Kampuchea or Cameroon or Cameroons or Cameron or Camerons or Cape Verde or Central African Republic or Chad or Chile or China or Colombia or Comoros or Comoro Islands or Comores or Mayotte or Congo or Zaire or Costa Rica or Cote d'Ivoire or Ivory Coast or Croatia or Cuba or Cyprus or Czechoslovakia

	or Czech Republic or Slovakia or Slovak Republic or Djibouti or French Somaliland or Dominica or Dominican Republic or East Timor or East Timur or Timor Leste or Ecuador or Egypt or United Arab Republic or El Salvador or Eritrea or Estonia or Ethiopia or Fiji or Gabon or Gabonese Republic or Gambia or Gaza or Georgia Republic or Georgian Republic or Ghana or Gold Coast or Greece or Grenada or Guatemala or Guinea or Guam or Guiana or Guyana or Haiti or Honduras or Hungary or India or Maldives or Indonesia or Iran or Iraq or Isle of Man or Jamaica or Jordan or Kazakhstan or Kazakh or Kenya or Kiribati or Korea or Kosovo or Kyrgyzstan or Kirghizia or Kyrgyz Republic or Kirghiz or Kirgizstan or Lao PDR or Laos or Latvia or Lebanon or Lesotho or Basutoland or Liberia or Libya or Lithuania or Macedonia or Madagascar or Malagasy Republic or Malaysia or Malaya or Malay or Sabah or Sarawak or Malawi or Nyasaland or Mali or Malta or Marshall Islands or Mauritania or Mauritius or Agalega Islands or Mexico or Micronesia or Middle East or Moldova or Moldovia or Moldovian or Mongolia or Montenegro or Morocco or Ifni or Mozambique or Myanmar or Myanma or Burma or Namibia or Nepal or Netherlands Antilles or New Caledonia or Nicaragua or Niger or Nigeria or Northern Mariana Islands or Oman or Muscat or Pakistan or Palau or Palestine or Panama or Paraguay or Peru or Philippines or Philipines or Phillipines or Phillippines or Poland or Portugal or Puerto Rico or Romania or Rumania or Roumania or Russia or Russian or Rwanda or Ruanda or Saint Kitts or St Kitts or Nevis or Saint Lucia or St Lucia or Saint Vincent or St Vincent or Grenadines or Samoa or Samoan Islands or Navigator Island or Navigator Islands or Sao Tome or Saudi Arabia or Senegal or Serbia or Montenegro or Seychelles or Sierra Leone or Slovenia or Sri Lanka or Ceylon or Solomon Islands or Somalia or Sudan or Suriname or Surinam or Swaziland or Syria or Tajikistan or Tadzhikistan or Tadjikistan or Tadzhik or Tanzania or Thailand or Togo or Togolese Republic or Tonga or Trinidad or Tobago or Tunisia or Turkey or Turkmenistan or Turkmen or Uganda or Ukraine or Uruguay or USSR or Soviet Union or Union of Soviet Socialist Republics or Uzbekistan or Uzbek or Vanuatu or New Hebrides or Venezuela or Vietnam or Viet Nam or West Bank or Yemen or Yugoslavia or Zambia or Zimbabwe or Rhodesia).hw,kf,ti,ab,cp.
58	((developing or less* developed or under developed or underdeveloped or middle income or low* income or underserved or under served or deprived or poor*) adj (countr* or nation? or population? or world)).ti,ab.
59	((developing or less* developed or under developed or underdeveloped or middle income or low* income) adj (economy or economies)).ti,ab.
60	(low* adj (gdp or gnp or gross domestic or gross national)).ti,ab.
61	(low adj3 middle adj3 countr*).ti,ab.
62	(lmic or lmics or third world or lami countr*).ti,ab.
63	transitional countr*.ti,ab.
64	55 or 56 or 57 or 58 or 59 or 60 or 61 or 62 or 63
65	53 and 64
66	53 not 65
67	52 not 53

PAIS International & EconLIT

Set#	Searched for
S1	ti(obes* OR overweight OR fat) AND ti(tax*)
S2	AB(food* or beverage* or soda or sodas or soft drink* or carbonated drink* or sugar* or fat or fats or sucrose or candy or sweet* or snack* or fastfood* or junkfood* or vending machine*)
S3	AB(tax or taxes or taxation or taxing or price or prices or pricing or subsidy or subsidies or incentiv* or voucher* or reward*)
S4	AB(elastic* NEAR/3 (price* or pricing or demand* or fiscal* or economic* or financ* or cost*))
S5	AB(policy NEAR/3 (price* or pricing or demand* or fiscal* or economic* or financ* or cost*))
S6	AB(policies NEAR/3 (price* or pricing or demand* or fiscal* or economic* or financ* or cost*))
S7	S3 OR S4 OR S5 OR S6
S8	body mass or body fat* or bmi or obes* or overweight or body weight
S10	(health* NEAR/3 (food* or eating or choice* or lifestyle or lifestyle))
S11	fruit* or vegetable*
S12	public health or healthy living or health promotion
S13	(food* NEAR/3 (consumption OR consumed OR intake))
S14	(energy* NEAR/3 (consumption OR consumed OR intake))
S15	(beverage* NEAR/3 (consumption OR consumed OR intake))
S16	(drink*2 NEAR/3 (consumption OR consumed OR intake))
S17	S8 OR S10 OR S11 OR S12 OR S13 OR S14 OR S15 OR S16

S18	S2 AND S7 AND S17
S21	ti(food* OR beverage* OR soda OR sodas OR soft drink* OR carbonated drink* OR sugar* OR fat OR fats OR sucrose OR candy OR sweet* OR snack* OR fastfood* OR junkfood* OR vending machine*)
S22	ti(tax or taxes or taxation or taxing or price or prices or pricing or subsidy or subsidies or incentiv* or voucher* or reward*)
S23	S21 AND S22
S24	S1 OR S18 OR S23
S25	(ti(Africa OR Caribbean OR West Indies OR South America OR Latin America OR Central America) OR ab(Africa OR Caribbean OR West Indies OR South America OR Latin America OR Central America) OR su(Africa OR Caribbean OR West Indies OR South America OR Latin America OR Central America)) OR ((developing NEAR/2 (countr* OR nation*2 OR population*2 OR world)) OR (less developed NEAR/2 (countr* OR nation*2 OR population*2 OR world)) OR (under developed NEAR/2 (countr* OR nation*2 OR population*2 OR world)) OR (underdeveloped NEAR/2 (countr* OR nation*2 OR population*2 OR world)) OR (middle income NEAR/2 (countr* OR nation*2 OR population*2 OR world)) OR (low income NEAR/2 (countr* OR nation*2 OR population*2 OR world)) OR (underserved NEAR/2 (countr* OR nation*2 OR population*2 OR world)) OR (under served NEAR/2 (countr* OR nation*2 OR population*2 OR world)) OR (deprived NEAR/2 (countr* OR nation*2 OR population*2 OR world)) OR (poor* NEAR/2 (countr* OR nation*2 OR population*2 OR world))) OR ((developing NEAR/2 (economy OR economies)) OR (less developed NEAR/2 (economy OR economies)) OR (under developed NEAR/2 (economy OR economies)) OR (underdeveloped NEAR/2 (economy OR economies)) OR (middle income NEAR/2 (economy OR economies)) OR (low income NEAR/2 (economy OR economies)) OR (underserved NEAR/2 (economy OR economies)) OR (under served NEAR/2 (economy OR economies)) OR (deprived NEAR/2 (economy OR economies)) OR (poor* NEAR/2 (economy OR economies))) OR (lmic OR lmics OR third world OR lami countr* OR transitional countr*) OR (Afghanistan OR Albania OR Algeria OR Angola OR Antigua OR Barbuda OR Argentina OR Armenia OR Armenian OR Aruba OR Azerbaijan OR Bahrain OR Bangladesh OR Barbados OR Benin OR Byelarus OR Byelorussian OR Belarus OR Belorussian OR Belorussia OR Belize OR Bhutan OR Bolivia OR Bosnia OR Herzegovina OR Hercegovina OR Botswana OR Brazil OR Brasil OR Bulgaria OR

	Burkina Faso OR Burkina Fasso OR Upper Volta OR Burundi OR Urundi OR Cambodia OR Khmer Republic OR Kampuchea OR Cameroon OR Cameroons OR Cameron OR Camerons OR Cape Verde OR Central African Republic OR Chad OR Chile OR China OR Colombia OR Comoros OR Comoro Islands OR Comores OR Mayotte OR Congo OR Zaire OR Costa Rica OR Cote d'Ivoire OR Ivory Coast OR Croatia OR Cuba OR Cyprus OR Czechoslovakia OR Czech Republic OR Slovakia OR Slovak Republic OR Djibouti OR French Somaliland OR Dominica OR Dominican Republic OR East Timor OR East Timur OR Timor Leste OR Ecuador OR Egypt OR United Arab Republic OR El Salvador OR Eritrea OR Estonia OR Ethiopia OR Fiji OR Gabon OR Gabonese Republic OR Gambia OR Gaza OR Georgia Republic OR Georgian Republic OR Ghana OR Gold Coast OR Greece OR Grenada OR Guatemala OR Guinea OR Guam OR Guiana OR Guyana OR Haiti OR Honduras OR Hungary OR India OR Maldives OR Indonesia OR Iran OR Iraq OR Isle of Man OR Jamaica OR Jordan OR Kazakhstan OR Kazakh OR Kenya OR Kiribati OR Korea OR Kosovo OR Kyrgyzstan OR Kirghizia OR Kyrgyz Republic OR Kirghiz OR Kirgizstan OR Lao PDR OR Laos OR Latvia OR Lebanon OR Lesotho OR Basutoland OR Liberia OR Libya OR Lithuania OR Macedonia OR Madagascar OR Malagasy Republic OR Malaysia OR Malaya OR Malay OR Sabah OR Sarawak OR Malawi OR Nyasaland OR Mali OR Malta OR Marshall Islands OR Mauritania OR Mauritius OR Agalega Islands OR Mexico OR Micronesia OR Middle East OR Moldova OR Moldovia OR Moldovian OR Mongolia OR Montenegro OR Morocco OR Ifni OR Mozambique OR Myanmar OR Myanma OR Burma OR Namibia OR Nepal OR Netherlands Antilles OR New Caledonia OR Nicaragua OR Niger OR Nigeria OR Northern Mariana Islands OR Oman OR Muscat OR Pakistan OR Palau OR Palestine OR Panama OR Paraguay OR Peru OR Philippines OR Philipines OR Phillipines OR Phillippines OR Poland OR Portugal OR Puerto Rico OR Romania OR Rumania OR Roumania OR Russia OR Russian OR Rwanda OR Ruanda OR Saint Kitts OR St Kitts OR Nevis OR Saint Lucia OR St Lucia OR Saint Vincent OR St Vincent OR Grenadines OR Samoa OR Samoan Islands OR Navigator Island OR Navigator Islands OR Sao Tome OR Saudi Arabia OR Senegal OR Serbia OR Montenegro OR Seychelles OR Sierra Leone OR Slovenia OR Sri Lanka OR Ceylon OR Solomon Islands OR Somalia OR Sudan OR Suriname OR Surinam OR Swaziland OR Syria OR Tajikistan OR Tadzhikistan OR Tadjikistan OR Tadzhik OR Tanzania OR Thailand OR Togo OR Togolese Republic OR Tonga OR Trinidad OR Tobago OR Tunisia OR Turkey OR Turkmenistan OR Turkmen OR Uganda OR Ukraine OR Uruguay OR USSR OR Soviet Union OR Union of Soviet Socialist Republics OR Uzbekistan OR Uzbek OR Vanuatu OR New Hebrides OR Venezuela OR Vietnam OR Viet Nam OR West Bank OR

	Yemen OR Yugoslavia OR Zambia OR Zimbabwe OR Rhodesia)
S26	S24 AND S25

Proquest Dissertations & Theses:

S7 S5 and S6

S6 S1 or S4

S5 (ti(Africa OR Caribbean OR West Indies OR South America OR Latin America OR Central America) OR ab(Africa OR Caribbean OR West Indies OR South America OR Latin America OR Central America) OR su(Africa OR Caribbean OR West Indies OR South America OR Latin America OR Central America)) OR ((developing NEAR/2 (countr* OR nation*2 OR population*2 OR world)) OR (less developed NEAR/2 (countr* OR nation*2 OR population*2 OR world)) OR (under developed NEAR/2 (countr* OR nation*2 OR population*2 OR world)) OR (underdeveloped NEAR/2 (countr* OR nation*2 OR population*2 OR world)) OR (middle income NEAR/2 (countr* OR nation*2 OR population*2 OR world)) OR (low income NEAR/2 (countr* OR nation*2 OR population*2 OR world)) OR (underserved NEAR/2 (countr* OR nation*2 OR population*2 OR world)) OR (under served NEAR/2 (countr* OR nation*2 OR population*2 OR world)) OR (deprived NEAR/2 (countr* OR nation*2 OR population*2 OR world)) OR (poor* NEAR/2 (countr* OR nation*2 OR population*2 OR world))) OR ((developing NEAR/2 (economy OR economies)) OR (less developed NEAR/2 (economy OR economies)) OR (under developed NEAR/2 (economy OR economies)) OR (underdeveloped NEAR/2 (economy OR economies)) OR (middle income NEAR/2 (economy OR economies)) OR (low income NEAR/2 (economy OR economies)) OR (underserved NEAR/2 (economy OR economies)) OR (under served NEAR/2 (economy OR economies)) OR (deprived NEAR/2 (economy OR economies)) OR (poor* NEAR/2 (economy OR economies))) OR (lmic OR lmics OR third world OR lami countr* OR transitional countr*) OR (Afghanistan OR Albania OR Algeria OR Angola OR Antigua OR Barbuda OR Argentina OR Armenia OR Armenian OR Aruba OR Azerbaijan OR Bahrain OR Bangladesh OR Barbados OR Benin OR Byelarus OR Byelorussian OR Belarus OR Belorussian OR Belorussia OR Belize OR Bhutan OR Bolivia OR Bosnia OR Herzegovina OR Hercegovina OR Botswana OR Brazil OR Brasil OR Bulgaria OR Burkina Faso OR Burkina Fasso OR Upper Volta OR Burundi OR Urundi OR Cambodia OR Khmer Republic OR Kampuchea OR Cameroon OR Cameroons OR Cameron OR Camerons OR Cape Verde OR Central African Republic OR Chad OR Chile OR China OR Colombia OR Comoros OR Comoro Islands OR Comores OR Mayotte OR Congo OR Zaire OR Costa Rica OR Cote d'Ivoire OR Ivory Coast OR Croatia OR Cuba OR Cyprus OR Czechoslovakia OR Czech Republic OR Slovakia OR Slovak Republic OR Djibouti OR French Somaliland OR Dominica OR Dominican Republic OR East Timor OR East Timur OR Timor Leste OR Ecuador OR Egypt OR United Arab Republic OR El Salvador OR Eritrea OR Estonia OR Ethiopia OR Fiji OR Gabon OR Gabonese Republic OR Gambia OR Gaza OR Georgia Republic OR Georgian Republic OR Ghana OR Gold Coast OR Greece OR Grenada OR Guatemala OR Guinea OR Guam OR Guiana OR Guyana OR Haiti OR Honduras OR Hungary OR India OR Maldives OR Indonesia OR Iran OR Iraq OR Isle of Man OR Jamaica OR Jordan OR Kazakhstan OR Kazakh OR Kenya OR Kiribati OR Korea OR Kosovo OR Kyrgyzstan OR Kirghizia OR Kyrgyz Republic OR Kirghiz OR Kirgizstan OR Lao PDR OR Laos OR Latvia OR Lebanon OR Lesotho OR Basutoland

OR Liberia OR Libya OR Lithuania OR Macedonia OR Madagascar OR Malagasy Republic OR Malaysia OR Malaya OR Malay OR Sabah OR Sarawak OR Malawi OR Nyasaland OR Mali OR Malta OR Marshall Islands OR Mauritania OR Mauritius OR Agalega Islands OR Mexico OR Micronesia OR Middle East OR Moldova OR Moldovia OR Moldovian OR Mongolia OR Montenegro OR Morocco OR Ifni OR Mozambique OR Myanmar OR Myanma OR Burma OR Namibia OR Nepal OR Netherlands Antilles OR New Caledonia OR Nicaragua OR Niger OR Nigeria OR Northern Mariana Islands OR Oman OR Muscat OR Pakistan OR Palau OR Palestine OR Panama OR Paraguay OR Peru OR Philippines OR Philipines OR Phillipines OR Phillippines OR Poland OR Portugal OR Puerto Rico OR Romania OR Rumania OR Roumania OR Russia OR Russian OR Rwanda OR Ruanda OR Saint Kitts OR St Kitts OR Nevis OR Saint Lucia OR St Lucia OR Saint Vincent OR St Vincent OR Grenadines OR Samoa OR Samoan Islands OR Navigator Island OR Navigator Islands OR Sao Tome OR Saudi Arabia OR Senegal OR Serbia OR Montenegro OR Seychelles OR Sierra Leone OR Slovenia OR Sri Lanka OR Ceylon OR Solomon Islands OR Somalia OR Sudan OR Suriname OR Surinam OR Swaziland OR Syria OR Tajikistan OR Tadzhikistan OR TNEAR/2ikistan OR Tadzhik OR Tanzania OR Thailand OR Togo OR Togolese Republic OR Tonga OR Trinidad OR Tobago OR Tunisia OR Turkey OR Turkmenistan OR Turkmen OR Uganda OR Ukraine OR Uruguay OR USSR OR Soviet Union OR Union of Soviet Socialist Republics OR Uzbekistan OR Uzbek OR Vanuatu OR New Hebrides OR Venezuela OR Vietnam OR Viet Nam OR West Bank OR Yemen OR Yugoslavia OR Zambia OR Zimbabwe OR Rhodesia)

S4 2 and 3

S3 ti(tax or taxes or taxation or taxing or price or prices or pricing or subsidy or subsidies or incentiv* or voucher* or reward*)

S2 ti(food* OR beverage* OR soda OR sodas OR soft drink* OR carbonated drink* OR sugar* OR fat OR fats OR sucrose OR candy OR sweet* OR snack* OR fastfood* OR junkfood* OR vending machine*)

S1 ti(obes* OR overweight OR fat) AND ti(tax*)

Science Citation Index, Social Science Citation Index, Conference Proceedings Citation Index – Science & Conference Prceedings Citation Index – Social Science & Humanities. (Web of Science, 1945-present)

20 #10 NOT #11

19 #11 NOT #18

18 #17 AND #11

17 #16 OR #15 OR #14 OR #13 OR #12

16 TS=(Afghanistan OR Albania OR Algeria OR Angola OR Antigua OR Barbuda OR Argentina OR Armenia OR Armenian OR Aruba OR Azerbaijan OR Bahrain OR Bangladesh OR Barbados OR Benin OR Byelarus OR Byelorussian OR Belarus OR Belorussian OR Belorussia OR Belize OR Bhutan OR Bolivia OR Bosnia OR Herzegovina OR Hercegovina OR Botswana OR Brazil OR Brasil OR Bulgaria OR Burkina Faso OR Burkina Fasso OR Upper Volta OR Burundi OR Urundi OR Cambodia OR Khmer Republic OR Kampuchea OR Cameroon OR Cameroons OR Cameron OR Camerons OR Cape Verde OR Central African Republic OR Chad OR Chile OR China OR Colombia OR Comoros OR Comoro Islands OR Comores OR Mayotte OR Congo OR Zaire OR Costa Rica OR Cote d'Ivoire OR Ivory Coast OR Croatia OR Cuba OR Cyprus OR Czechoslovakia OR Czech Republic OR Slovakia OR Slovak Republic OR Djibouti OR French Somaliland OR Dominica OR Dominican Republic OR East Timor OR East Timur OR Timor Leste OR Ecuador OR Egypt OR United Arab Republic OR El Salvador OR Eritrea OR Estonia OR Ethiopia OR Fiji OR Gabon OR Gabonese Republic OR Gambia OR Gaza OR Georgia Republic OR Georgian Republic OR Ghana OR Gold Coast OR Greece OR Grenada OR Guatemala OR Guinea OR Guam OR Guiana OR Guyana OR Haiti OR Honduras OR Hungary OR India OR Maldives OR Indonesia OR Iran OR Iraq OR Isle of Man OR Jamaica OR Jordan OR Kazakhstan OR Kazakh OR Kenya OR Kiribati OR Korea OR Kosovo OR Kyrgyzstan OR Kirghizia OR Kyrgyz Republic OR Kirghiz OR Kirgizstan OR Lao PDR OR Laos OR Latvia OR Lebanon OR Lesotho OR Basutoland OR Liberia OR Libya OR Lithuania OR Macedonia OR Madagascar OR Malagasy Republic OR Malaysia OR Malaya OR Malay OR Sabah OR Sarawak OR Malawi OR Nyasaland OR Mali OR Malta OR Marshall Islands OR Mauritania OR Mauritius OR Agalega Islands OR Mexico OR Micronesia OR Middle East OR Moldova OR Moldovia OR Moldovian OR Mongolia OR Montenegro OR Morocco OR Ifni OR Mozambique OR Myanmar OR Myanma OR Burma OR Namibia OR Nepal OR Netherlands Antilles OR New Caledonia OR Nicaragua OR Niger OR Nigeria OR Northern Mariana Islands OR Oman OR Muscat OR Pakistan OR Palau OR Palestine OR Panama OR Paraguay OR Peru OR Philippines OR Philipines OR Phillipines OR Phillippines OR Poland OR Portugal OR Puerto Rico OR Romania OR Rumania OR Roumania OR Russia OR Russian OR Rwanda OR Ruanda OR Saint Kitts OR St Kitts OR Nevis OR Saint Lucia OR St Lucia OR Saint Vincent OR St Vincent OR Grenadines OR Samoa OR Samoan Islands OR Navigator Island OR Navigator Islands OR Sao Tome OR Saudi Arabia OR Senegal OR Serbia OR Montenegro OR Seychelles OR Sierra Leone OR Slovenia OR Sri Lanka OR Ceylon OR Solomon Islands OR Somalia OR Sudan OR Suriname OR Surinam OR Swaziland OR Syria OR Tajikistan OR Tadzhikistan OR TNEAR/2ikistan OR Tadzhik OR Tanzania OR Thailand OR Togo OR Togolese Republic OR Tonga OR Trinidad OR Tobago OR Tunisia OR Turkey OR

Turkmenistan OR Turkmen OR Uganda OR Ukraine OR Uruguay OR USSR OR Soviet Union OR Union of Soviet Socialist Republics OR Uzbekistan OR Uzbek OR Vanuatu OR New Hebrides OR Venezuela OR Vietnam OR Viet Nam OR West Bank OR Yemen OR Yugoslavia OR Zambia OR Zimbabwe OR Rhodesia) OR AD=(Afghanistan OR Albania OR Algeria OR Angola OR Antigua OR Barbuda OR Argentina OR Armenia OR Armenian OR Aruba OR Azerbaijan OR Bahrain OR Bangladesh OR Barbados OR Benin OR Byelarus OR Byelorussian OR Belarus OR Belorussian OR Belorussia OR Belize OR Bhutan OR Bolivia OR Bosnia OR Herzegovina OR Hercegovina OR Botswana OR Brazil OR Brasil OR Bulgaria OR Burkina Faso OR Burkina Fasso OR Upper Volta OR Burundi OR Urundi OR Cambodia OR Khmer Republic OR Kampuchea OR Cameroon OR Cameroons OR Cameron OR Camerons OR Cape Verde OR Central African Republic OR Chad OR Chile OR China OR Colombia OR Comoros OR Comoro Islands OR Comores OR Mayotte OR Congo OR Zaire OR Costa Rica OR Cote d'Ivoire OR Ivory Coast OR Croatia OR Cuba OR Cyprus OR Czechoslovakia OR Czech Republic OR Slovakia OR Slovak Republic OR Djibouti OR French Somaliland OR Dominica OR Dominican Republic OR East Timor OR East Timur OR Timor Leste OR Ecuador OR Egypt OR United Arab Republic OR El Salvador OR Eritrea OR Estonia OR Ethiopia OR Fiji OR Gabon OR Gabonese Republic OR Gambia OR Gaza OR Georgia Republic OR Georgian Republic OR Ghana OR Gold Coast OR Greece OR Grenada OR Guatemala OR Guinea OR Guam OR Guiana OR Guyana OR Haiti OR Honduras OR Hungary OR India OR Maldives OR Indonesia OR Iran OR Iraq OR Isle of Man OR Jamaica OR Jordan OR Kazakhstan OR Kazakh OR Kenya OR Kiribati OR Korea OR Kosovo OR Kyrgyzstan OR Kirghizia OR Kyrgyz Republic OR Kirghiz OR Kirgizstan OR Lao PDR OR Laos OR Latvia OR Lebanon OR Lesotho OR Basutoland OR Liberia OR Libya OR Lithuania OR Macedonia OR Madagascar OR Malagasy Republic OR Malaysia OR Malaya OR Malay OR Sabah OR Sarawak OR Malawi OR Nyasaland OR Mali OR Malta OR Marshall Islands OR Mauritania OR Mauritius OR Agalega Islands OR Mexico OR Micronesia OR Middle East OR Moldova OR Moldovia OR Moldovian OR Mongolia OR Montenegro OR Morocco OR Ifni OR Mozambique OR Myanmar OR Myanma OR Burma OR Namibia OR Nepal OR Netherlands Antilles OR New Caledonia OR Nicaragua OR Niger OR Nigeria OR Northern Mariana Islands OR Oman OR Muscat OR Pakistan OR Palau OR Palestine OR Panama OR Paraguay OR Peru OR Philippines OR Philipines OR Phillipines OR Phillippines OR Poland OR Portugal OR Puerto Rico OR Romania OR Rumania OR Roumania OR Russia OR Russian OR Rwanda OR Ruanda OR Saint Kitts OR St Kitts OR Nevis OR Saint Lucia OR St Lucia OR Saint Vincent OR St Vincent OR Grenadines OR Samoa OR Samoan Islands OR Navigator Island OR Navigator Islands OR Sao Tome OR Saudi Arabia OR Senegal OR Serbia OR Montenegro OR Seychelles OR Sierra Leone OR Slovenia OR Sri Lanka OR Ceylon OR Solomon Islands OR Somalia OR Sudan OR Suriname OR Surinam OR Swaziland OR Syria OR Tajikistan OR Tadzhikistan OR TNEAR/2ikistan OR Tadzhik OR Tanzania OR Thailand OR Togo OR Togolese Republic OR Tonga OR Trinidad OR Tobago OR Tunisia OR Turkey OR Turkmenistan OR Turkmen OR Uganda OR Ukraine OR Uruguay OR USSR OR Soviet Union OR Union of Soviet Socialist Republics OR Uzbekistan OR Uzbek OR Vanuatu OR New Hebrides OR Venezuela OR Vietnam OR Viet Nam OR West Bank OR Yemen OR Yugoslavia OR Zambia OR Zimbabwe OR Rhodesia)

15 TS=(lmic OR lmics OR "third world" OR "lami countr*" OR "transitional countr*")

14 TS=((developing NEAR/2 (economy OR economies))) OR TS=(("less developed" NEAR/2 (economy OR economies))) OR TS=(("under developed" NEAR/2 (economy OR economies))) OR

TS=((underdeveloped NEAR/2 (economy OR economies))) OR TS=(("middle income" NEAR/2 (economy OR economies))) OR TS=(("low income" NEAR/2 (economy OR economies))) OR TS=((underserved NEAR/2 (economy OR economies))) OR TS=(("under served" NEAR/2 (economy OR economies))) OR TS=((deprived NEAR/2 (economy OR economies))) OR TS=((poor* NEAR/2 (economy OR economies)))

13 TS=((developing NEAR/2 (countr* OR nation*2 OR population*2 OR world))) OR TS=(("less developed" NEAR/2 (countr* OR nation*2 OR population*2 OR world))) OR TS=(("under developed" NEAR/2 (countr* OR nation*2 OR population*2 OR world))) OR TS=((underdeveloped NEAR/2 (countr* OR nation*2 OR population*2 OR world))) OR TS=(("middle income" NEAR/2 (countr* OR nation*2 OR population*2 OR world))) OR TS=(("low income" NEAR/2 (countr* OR nation*2 OR population*2 OR world))) OR TS=((underserved NEAR/2 (countr* OR nation*2 OR population*2 OR world))) OR TS=(("under served" NEAR/2 (countr* OR nation*2 OR population*2 OR world))) OR TS=((deprived NEAR/2 (countr* OR nation*2 OR population*2 OR world))) OR TS=((poor* NEAR/2 (countr* OR nation*2 OR population*2 OR world)))

12 TS=(Africa OR Caribbean OR "West Indies" OR "South America" OR "Latin America" OR "Central America") OR AD=(Africa OR Caribbean OR "West Indies" OR "South America" OR "Latin America" OR "Central America")

11 #9 OR #5 OR #4 OR #3 OR #2 OR #1 Refined by: Languages=(ENGLISH)

10 #9 OR #5 OR #4 OR #3 OR #2 OR #1

9 #8 AND #7 AND #6

8 TS=("body mass" OR bmi OR "body weight" OR obes* OR overweight OR "public health" OR "healthy living" OR "health promotion") OR TS=("public health" or "healthy living" or "health promotion") OR TS=(fruit* or vegetable*) OR TS=((health* NEAR/3 (food* or eating or choice* or lifestyle or lifestyle))) OR TS=((food* NEAR/3 (consumption OR consumed OR intake))) OR TS=((energy* NEAR/3 (consumption OR consumed OR intake))) OR TS=((beverage* NEAR/3 (consumption OR consumed OR intake))) OR TS=((drink*2 NEAR/3 (consumption OR consumed OR intake)))

7 TS=((elastic* NEAR/3 (price* or pricing or demand* or fiscal* or economic* or financ* or cost*))) OR TS=((policy NEAR/3 (price* or pricing or demand* or fiscal* or economic* or financ* or cost*))) OR TS=((policies NEAR/3 (price* or pricing or demand* or fiscal* or economic* or financ* or cost*))) OR TS=(tax or taxes or taxation or taxing or subsidy or subsidies or incentiv* or voucher*)

6 TS=(food* or beverage* or soda or sodas or soft drink* or carbonated drink* or sugar* or fat or fats or sucrose or candy or sweet* or snack* or fastfood* or junkfood* or vending machine*)

5 TI=((obes* OR overweight OR fat)) AND TI=(tax OR taxes OR taxation OR taxing)

4 TI=(tax or taxes or taxation or taxing or price or prices or pricing or subsidy or subsidies or incentiv* or voucher*) AND TI=(food* OR beverage* OR soda OR sodas OR "soft drink*" OR "carbonated drink*" OR sugar* OR fat OR fats OR sucrose OR candy OR sweet* OR snack* OR

fastfood* OR junkfood* OR "vending machine*") AND TS=(fruit* OR vegetable)

3 TI=(tax or taxes or taxation or taxing or price or prices or pricing or subsidy or subsidies or incentiv* or voucher*) AND TI=(food* OR beverage* OR soda OR sodas OR "soft drink*" OR "carbonated drink*" OR sugar* OR fat OR fats OR sucrose OR candy OR sweet* OR snack* OR fastfood* OR junkfood* OR "vending machine*") AND TS=((health* NEAR/3 (food* or eating or choice* or lifestyle or lifestyle)))

2 TI=(tax or taxes or taxation or taxing or price or prices or pricing or subsidy or subsidies or incentiv* or voucher*) AND TI=(food* OR beverage* OR soda OR sodas OR "soft drink*" OR "carbonated drink*" OR sugar* OR fat OR fats OR sucrose OR candy OR sweet* OR snack* OR fastfood* OR junkfood* OR "vending machine*") AND TS=("body mass" OR bmi OR "body weight" OR obes* OR overweight OR "public health" OR "healthy living" OR "health promotion")

1 TI=(tax or taxes or taxation or taxing or subsidy or subsidies or incentiv* or voucher*) AND TI=(food* OR beverage* OR soda OR sodas OR "soft drink*" OR "carbonated drink*" OR sugar* OR fat OR fats OR sucrose OR candy OR sweet* OR snack* OR fastfood* OR junkfood* OR "vending machine*")

Grey literature:

OECDiLibrary http://www.oecd-ilibrary.org/ - 20 results

(food* OR beverage* OR soda OR sodas OR soft drink* OR carbonated drink* OR sugar* OR fat OR fats OR sucrose OR candy OR sweet* OR snack* OR fastfood* OR junkfood* OR vending machine*) AND (tax OR taxes OR taxation OR taxing OR subsidy OR subsidies OR incentiv* OR voucher*) – in Title and abstract

(obese OR obesity OR overweight OR fat OR "healthy eating" OR "healthy lifestyle") AND (tax OR taxes OR taxation OR taxing OR subsidy OR subsidies OR incentiv* OR voucher*) – in Title

(food* OR beverage* OR soda OR sodas OR soft drink* OR carbonated drink* OR sugar* OR fat OR fats OR sucrose OR candy OR sweet* OR snack* OR fastfood* OR junkfood* OR vending machine*) AND ("price elasticity" OR "price elasticities" OR "deman elasticity" OR "demand elasticities" OR "financial elasticity" OR "financial elasticities" OR "pricing elasticity" OR "pricing elasticities" OR "economic elasticity" OR "economic elasticities" OR "cost elasticity" OR "cost elasticities" OR "fiscal elasticity" OR "fiscal elasticities") – in Title and abstract

ClinicalTrials.gov http://clinicaltrials.gov – 10 results

Condition=food* OR beverage* OR soda OR sodas OR soft drink* OR carbonated drink* OR sugar* OR fat OR fats OR sucrose OR candy OR sweet* OR snack* OR fastfood* OR junkfood* OR vending machine* AND Intervention= tax OR taxes OR taxation OR taxing OR subsidy OR subsidies OR incentiv* OR voucher*

Title=food* OR beverage* OR soda OR sodas OR soft drink* OR carbonated drink* OR sugar* OR fat OR fats OR sucrose OR candy OR sweet* OR snack* OR fastfood* OR junkfood* OR vending machine* AND Intervention= tax OR taxes OR taxation OR taxing OR subsidy OR subsidies OR incentiv* OR voucher*

Condition=overweight OR obese OR obesity AND intervention= tax OR taxes OR taxation OR taxing OR subsidy OR subsidies OR incentiv* OR voucher*

Condition=overweight OR obese OR obesity AND title= tax OR taxes OR taxation OR taxing OR subsidy OR subsidies OR incentiv* OR voucher*

Title=overweight OR obese OR obesity AND intervention= tax OR taxes OR taxation OR taxing OR subsidy OR subsidies OR incentiv* OR voucher*

OPENGrey http://www.opengrey.eu/ - 0 results

"fat tax", "fat taxes" , "soda tax" , "soda taxes" , "soft drinks tax" , "soft drinks taxes" , "sugar tax" , "sugar taxes" , "fastfood tax" , "fastfood taxes" , "fast food tax" , "fast food taxes" , “junkfood tax” , “junk food tax” , “junkfood taxes” , “junk food taxes”, “food subsidy”, “food subsidies”, food incentive, food incentives

Web searches on Google: done by running the following searches and then browsed the 1st 2-3 pages of results to see if there is anything relevant.

General web searches:

("fat tax" OR "fat taxes" OR "soda tax" OR "soda taxes" OR "soft drinks tax" OR "soft drinks taxes" OR "sugar tax" OR "sugar taxes" OR "fastfood tax" OR "fastfood taxes" OR "fast food tax" OR "fast food taxes" OR “junkfood tax” OR “junk food tax” OR “junkfood taxes” OR “junk food taxes”)

("food tax" OR "food taxes" OR "food taxation") AND (obese OR obesity OR overweight)

("fiscal policy" OR "fiscal policies" OR "financial policy" OR "financial policies" OR "economic policy" OR "economic policies) AND (obese OR obesity OR overweight)

Using Google to search specific web-sites: OECD, World Bank, WHO, .org sites & .org.uk sites. You may be able to think of other organisations.

("fat tax" OR "fat taxes" OR "soda tax" OR "soda taxes" OR "soft drinks tax" OR "soft drinks taxes" OR "sugar tax" OR "sugar taxes" OR "fastfood tax" OR "fastfood taxes" OR "fast food tax" OR "fast food taxes" OR “junkfood tax” OR “junk food tax” OR “junkfood taxes” OR “junk food taxes”) site:who.int

("food tax" OR "food taxes" OR "food taxation") AND (obese OR obesity OR overweight) site:who.int

("fiscal policy" OR "fiscal policies" OR "financial policy" OR "financial policies" OR "economic policy" OR "economic policies) AND (obese OR obesity OR overweight) site:who.int

("fat tax" OR "fat taxes" OR "soda tax" OR "soda taxes" OR "soft drinks tax" OR "soft drinks taxes" OR "sugar tax" OR "sugar taxes" OR "fastfood tax" OR "fastfood taxes" OR "fast food tax" OR "fast food taxes" OR “junkfood tax” OR “junk food tax” OR “junkfood taxes” OR “junk food taxes”) site:oecd.org

("food tax" OR "food taxes" OR "food taxation") AND (obese OR obesity OR overweight) site:oecd.org

("fiscal policy" OR "fiscal policies" OR "financial policy" OR "financial policies" OR "economic policy" OR "economic policies) AND (obese OR obesity OR overweight) site:oecd.org
("fat tax" OR "fat taxes" OR "soda tax" OR "soda taxes" OR "soft drinks tax" OR "soft drinks taxes" OR "sugar tax" OR "sugar taxes" OR "fastfood tax" OR "fastfood taxes" OR "fast food tax" OR "fast food taxes" OR “junkfood tax” OR “junk food tax” OR “junkfood taxes” OR “junk food taxes”) site:worldbank.org

("food tax" OR "food taxes" OR "food taxation") AND (obese OR obesity OR overweight) site:worldbank.org

("fiscal policy" OR "fiscal policies" OR "financial policy" OR "financial policies" OR "economic policy" OR "economic policies) AND (obese OR obesity OR overweight) site:worldbank.org

("fat tax" OR "fat taxes" OR "soda tax" OR "soda taxes" OR "soft drinks tax" OR "soft drinks taxes" OR "sugar tax" OR "sugar taxes" OR "fastfood tax" OR "fastfood taxes" OR "fast food tax" OR "fast food taxes" OR “junkfood tax” OR “junk food tax” OR “junkfood taxes” OR “junk food taxes”) site:.org

("food tax" OR "food taxes" OR "food taxation") AND (obese OR obesity OR overweight) site:.org

("fiscal policy" OR "fiscal policies" OR "financial policy" OR "financial policies" OR "economic policy" OR "economic policies) AND (obese OR obesity OR overweight) site:.org

("fat tax" OR "fat taxes" OR "soda tax" OR "soda taxes" OR "soft drinks tax" OR "soft drinks taxes" OR "sugar tax" OR "sugar taxes" OR "fastfood tax" OR "fastfood taxes" OR "fast food tax" OR "fast food taxes" OR “junkfood tax” OR “junk food tax” OR “junkfood taxes” OR “junk food taxes”) site:org.uk

("food tax" OR "food taxes" OR "food taxation") AND (obese OR obesity OR overweight) site:org.uk

("fiscal policy" OR "fiscal policies" OR "financial policy" OR "financial policies" OR "economic policy" OR "economic policies) AND (obese OR obesity OR overweight) site:org.uk
