# Supplementary material for: Studying the consumption and health outcomes of fiscal interventions (taxes and subsidies) on food and beverages in countries of different income classifications; a systematic review
Source: BMC Public Health. 2015 Sep 14;15:887. doi: 10.1186/s12889-015-2201-8 (PMC4570679; doi:10.1186/s12889-015-2201-8)
Supplement: Additional file 2: — Adjusted criteria for the quality assessment tool for quantitative studies. (PDF 88 kb) [file 12889_2015_2201_MOESM2_ESM.pdf]

Additional file 2: Adjusted criteria for the quality assessment tool for quantitative studies.

The quality assessment tool for quantitative studies (Quality assessment Tool for quantitative studies from the Effective Public Health Practice Project -EPHPP-) [1] was developed to assess the quality of the observational and clinical studies based on study designs focused at the individual level. This tool was applied to the studies under review. In order to adapt the tool to the selected studies, several criteria were modified, as described below.

A) SELECTION BIAS

Selection bias is unlikely in national level studies.

Selection bias is somewhat likely in local level studies.

Selection bias likely when the study was targeted at specific members of the population.

B) STUDY DESIGN

No changes made.

C) CONFOUNDERS

If the study did not have a control group, the assessment was taken as not applicable (NA).

D) BLINDING

The answer to the first question (Q1) was 2 when the data was not linked to the intervention.

E) DATA COLLECTION METHODS

No changes made.

F) WITHDRAWALS AND DROP-OUTS

Withdrawals and dropouts do not occur when the total local or national level data collection was done.

Withdrawals and dropouts are possible when a nationally representative sample was surveyed.

Withdrawals and dropouts are possible when a sample was taken from the study population.

G) FINAL SCORING

When the final assessment of a component was not indicated in the dictionary as a possible answer, it was compared to the closest answers and the lower assessment (usually weak) was set, adopting a conservative decision.

References

1. *Effective Public Health Practice Project: Quality assessment Tool for Quantitative Studies.* http://www.ephpp.ca/tools.html [Accessed on 20 June 2015].
