# Supplementary material for: Studying the consumption and health outcomes of fiscal interventions (taxes and subsidies) on food and beverages in countries of different income classifications; a systematic review
Source: BMC Public Health. 2015 Sep 14;15:887. doi: 10.1186/s12889-015-2201-8 (PMC4570679; doi:10.1186/s12889-015-2201-8)
Supplement: Additional file 3: — Description of the intervention and quality assessment of study. (DOC 106 kb) [file 12889_2015_2201_MOESM3_ESM.doc]

| **Additional file 3. Description of intervention and quality assessment of study.** | | | | | | | | | | |
| --- | --- | --- | --- | --- | --- | --- | --- | --- | --- | --- |
| **Author and country** | **Description of Intervention** | **Selection bias** | **Study design** | **Control for confounders** | **Blinding** | **Data collection** | **Withdrawals and dropouts** | **Global rating** | | |
| Bahl  Ireland | Excise tax on soft drinks decreased from IR£ 0.37/gallon in 1980-1990 to IR£ 0.29/gallon in 1990-1992 | *** | * | NA | *** | *** | *** | Moderate | | |
| Oaks  USA | Intervention group had state tax of 5.5% on soft drinks and selected snacks. Control group had no tax on snacks | ** | ** | *** | *** | *** | *** | Strong | | |
| Kim  USA | State level taxes on soft drinks or snacks | ** | * | NA | *** | *** | *** | Moderate | | |
| Fletcher  USA | Mean soft drink tax rate among states with a tax between 4.1 -5.1% | ** | * | NA | * | *** | *** | Weak | | |
| Powell  USA | State-level carbonated soda sales tax range 0-8% | ** | * | NA | *** | *** | ** | Moderate | | |
| Fletcher  USA | State - level soft drink taxes. Range of mean total tax 3.3% – 5.0% | *** | * | NA | *** | *** | *** | Moderate | | |
| Fletcher  USA | Mean soft drink tax rate among states with a tax 4.724% | *** | * | *** | *** | *** | *** | Moderate | | |
| Nicholson  USA | State level fast food restaurant and soda taxes | ** | * | ** | *** | *** | *** | Moderate | | |
| Sturm  USA | State level carbonated soda sales tax is 4.2% | ** | * | NA | ** | *** | ** | Moderate | | |
| Currie  USA | Standard Food Stamp Programme (FSP) –monthly food vouchers for any foods up to $142 per households per month dependent on income. | *** | ** | NA | *** | *** | *** | Strong | | |
| Herman  USA | Standard WIC programme plus $10 voucher weekly for Fruit and Vegetables at two sites: 1. local supermarket (n=140), 2. farmer’s market (n=168). | * | ** | *** | * | *** | *** | Weak | | |
| Baum  USA | Food Stamps Programme on expectant mothers. | * | * | NA | * | *** | * | Weak | | |
| Black  Australia | Weekly box of subsidized fruit and vegetables up to $60 linked to preventive health services and nutritional promotion to children 0-17 years (n=167) from 55 families. Baseline and after 12 month assessment completed. | * | * | NA | *** | *** | * | Weak | | |
| Musgrove  Brazil | Evaluation of four food subsidy programmes. Two programmes distributed free foods while another two programmes subsidized four or more basic food stuffs. | * | * | * | * | ** | * | Weak | | |
| Sampaio  Brazil | Intervention group was given 20% food – price subsidies for 11 commodities. Comparison of birth weight was done with a control group. | * | ** | ** | * | ** | ** | Weak | | |
| Osberg  China | Food coupons for the purchase of rice, flour, and cooking oil at below market prices. The subsidy rate was 16.5% of the income of a three person family living at US$ 2 per day. 1991-1993 food subsidies were initially in place, 1993-2000 food subsidies had largely been abolished. | ** | * | * | ** | *** | * | Weak | | |
| An  South Africa | Up to 25%discount on selected food items in about 800 supermarkets. | * | ** | * | ** | *** | * | Weak | | |
| Asfaw  Egypt | Food subsidy programme: 57% for bread; 42–62% for sugar | * | * | NA | * | *** | * | Weak | | |
|  |  |  |  |  |  |  |  |  | |  |
| Quality assessment tool for quantitative studies (McMaster University): Effective public health practice project (EPHPP). When the assessment of a component was not indicated in the tool, the lower assessment (usually weak) was set.  *= weak; **= moderate; ***= strong. | | | | | | | | |  | |
| NA – Not Applicable | |  |  |  |  |  |  |  | |  |
